# Identification of Glycoprotein Biomarkers in Breast Cancer by MALDI

**DOI:** 10.3390/life16030498

**Published:** 2026-03-18

**Authors:** David Aebisher, Klaudia Dynarowicz, Izabela Rudy, Kacper Rogóż, Dorota Bartusik-Aebisher, Aleksandra Kawczyk-Krupka

**Affiliations:** 1 Department of Photomedicine and Physical Chemistry, Faculty of Medicine, University of Rzeszów, 35-310 Rzeszów, Poland; 2 Department of Biochemistry and General Chemistry, Faculty of Medicine, University of Rzeszów, 35-310 Rzeszów, Poland; kdynarowicz@ur.edu.pl (K.D.);; 3 Student Scientific Club of Biochemists URCell, Faculty of Medicine, University of Rzeszów, 35-959 Rzeszów, Poland; 4 Department of Internal Diseases, Angiology and Physical Medicine, Center for Laser Diagnostics and Therapy, Faculty of Medical Sciences in Zabrze, Medical University of Silesia, 40-055 Katowice, Poland

**Keywords:** breast cancer, glycoproteins, glycosylation, MALDI-TOF MS, MALDI imaging, biomarkers

## Abstract

Protein glycosylation plays a pivotal role in breast cancer biology, influencing cell proliferation, adhesion, migration, and immune evasion. Aberrant N- and O-glycosylation are hallmarks of neoplastic transformation and serve as sensitive indicators of disease progression. This review aims to characterize glycoprotein biomarkers in breast cancer identified using Matrix-Assisted Laser Desorption/Ionization (MALDI) Mass Spectrometry. We examine specific glycosylation alterations—including hypersialylation, fucosylation, and truncated O-glycans—across different molecular subtypes (Luminal A/B, HER2-positive, TNBC) and assess their diagnostic and prognostic potential. Methodologically, the review contrasts MALDI-based profiling and Imaging Mass Spectrometry (MALDI-IMS) with other proteomic techniques, highlighting MALDI’s advantages in throughput and spatial resolution alongside its technical limitations. Furthermore, we discuss emerging frontiers in the field, such as the shift from whole-serum analysis to “liquid biopsy” components (e.g., extracellular vesicles). Ultimately, we argue that implementing quantitative glycoproteomics is essential for advancing personalized oncology.

## 1. Introduction

Breast cancer remains one of the most frequently diagnosed malignancies in women worldwide and poses a significant clinical and biological challenge. This disease is characterized by both a high incidence and a variable clinical course, which impacts diagnostic, therapeutic, and prognostic options [1]. Despite significant progress in molecular classification and targeted therapies, the clinical management of breast cancer continues to face challenges related to tumor heterogeneity [2], variable treatment response [3], and the lack of sufficiently sensitive biomarkers for early detection and disease monitoring [4]. Consequently, the identification of reliable molecular signatures capable of reflecting tumor biology and predicting clinical outcomes remains a central objective in oncology research [5]. Among the diverse molecular changes observed in cancer, abnormal protein glycosylation has emerged as a particularly important feature of cancer progression [6]. Glycosylation is one of the most common post-translational modifications and plays a critical role in regulating protein folding, stability, receptor signaling, and cell–cell interactions [7,8]. In malignant cells, dysregulated glycosylation can alter adhesion properties, modulate immune recognition, and facilitate metastatic dissemination [9]. These changes are frequently reflected in the glycosylation patterns of circulating or membrane-associated glycoproteins, making them attractive candidates for cancer biomarker discovery [10,11,12]. Advances in mass spectrometry-based technologies have significantly expanded the possibilities for characterizing glycoproteins and their associated glycan structures. Techniques such as liquid chromatography-mass spectrometry (LC-MS) have enabled high-resolution molecular profiling, enabling the identification of numerous cancer-associated glycoproteins. However, translating these discoveries into clinically useful diagnostic tools remains challenging due to the complexity, cost, and limited throughput of some analytical platforms [13,14,15,16]. Matrix-assisted laser desorption/ionization mass spectrometry (MALDI-MS) offers several advantages that make it particularly attractive for translational biomarker research. MALDI platforms enable rapid spectral acquisition, relatively simple sample preparation, and high-throughput analysis, which is highly compatible with clinical laboratory workflows. Furthermore, MALDI imaging mass spectrometry enables spatial molecular profiling directly from tissue sections, providing unique insights into tumor heterogeneity and microenvironmental interactions. These features make MALDI-based glycoproteomic analysis a promising bridge between molecular discoveries and clinically applicable diagnostic strategies [17,18,19,20,21,22,23,24,25]. In this context, this review focuses on the contribution of MALDI-based technologies to the identification and characterization of glycoprotein biomarkers in breast cancer. Recent advances in MALDI-based glycoproteomic profiling are presented, and new applications in biomarker discovery and cancer biology are identified. Future directions for integrating MALDI-based approaches into translational oncology and precision medicine are also outlined.

## 2. MALDI Technology for Glycoprotein Identification

MALDI (Matrix-Assisted Laser Desorption/Ionization) technology plays a significant role in glycoproteomic research due to its ability to analyze complex biomolecules, such as glycans and glycoproteins, with minimal ion fragmentation. In the context of breast cancer research, this technique is particularly useful for analyzing glycosylation profiles and identifying changes characteristic of malignant transformation. MALDI allows for the analysis of both released glycans and glycopeptides, making it a versatile tool in biomarker research.

### 2.1. Principles of MALDI-TOF MS: Specificity for Glycan Analysis

**Figure 1:**
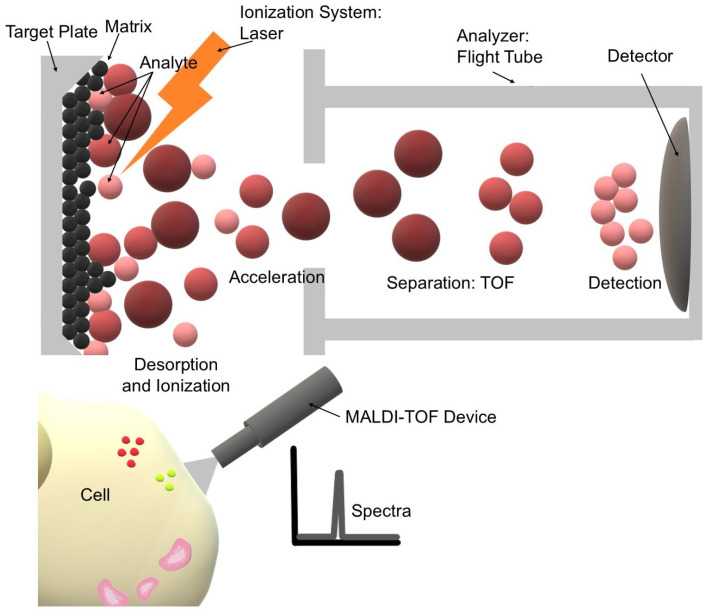
Schematic representation of the MALDI-TOF MS principle workflow specifically for glycan analysis. The process initiates with sample preparation, where the analyte (e.g., released glycans from breast cancer tissue or serum) is co-crystallized with an excess of UV-absorbing matrix on a conductive target plate. Upon irradiation with nanosecond laser pulses, the matrix absorbs energy, facilitating the rapid, “soft” desorption and ionization of intact analyte molecules into the gas phase, predominantly yielding singly charged ions. These ions are subsequently accelerated by an electric potential into a field-free high-vacuum flight tube. In the Time-of-Flight (TOF) analyzer, ions are separated based on their mass-to-charge ratio (*m*/*z*), as lighter ions traverse the drift path faster than heavier ones. The arrival times at the detector are recorded to generate a mass spectrum, providing a characteristic molecular profile of the sample (Created by the authors).

In the conventional vacuum MALDI technique, the sample is analyzed as a mixture of the analyte with a suitable organic matrix that absorbs the energy of the laser pulse. Upon irradiation, the analyte molecules undergo rapid desorption and ionization, occurring primarily through proton transfer from the matrix (Figure 1). A distinct physical advantage of MALDI in this context—compared to Electrospray Ionization (ESI)—is that it predominantly generates singly charged ions. While ESI often produces complex spectra with multiply charged species, MALDI ensures that each glycan structure typically corresponds to a single signal peak. This mechanism allows for rapid, precise, and interpretable molecular mass measurements, which are crucial in the analysis of heterogeneous glycan structures [26].

Crucially, the selection of MALDI over other proteomic techniques represents a strategic ‘clinical compromise. Although LC-MS/MS provides unparalleled structural depth and quantitative accuracy, its processes are generally labor-intensive and involve chromatographic separation steps that can somewhat limit rapid, high-throughput screening. MALDI-TOF MS occupies the unique ‘sweet spot’ between these extremes: it offers tolerance to biological matrices (salts, blood components) that would clog LC columns, and provides a speed of acquisition essential for analyzing large patient cohorts. Furthermore, unlike solution-based LC-MS, MALDI retains the unique potential for spatial mapping (Imaging MS), allowing glycosylation changes to be directly correlated with histopathological features—a capability that justifies its prioritization in biomarker discovery workflows [27,28].

The resulting ions are accelerated in an electric field and directed to a time-of-flight (TOF) analyzer, where their mass-to-charge ratio (*m*/*z*) is determined based on the time it takes to reach the detector. The TOF analyzer is characterized by a wide mass range and high resolution, enabling the simultaneous detection of multiple glycan and glycoprotein structures in a single measurement. This is particularly important in glycoproteomic studies, as glycans exhibit significant mass heterogeneity resulting from differences in monosaccharide composition and degree of branching. The use of a reflectron further improves the accuracy of mass measurements, enabling the distinction between closely related structures [29,30]. A comparative overview of MALDI-TOF MS capabilities versus other analytical techniques, such as LC-ESI-MS and Lectin Microarrays, is presented in Table 1.

One of the main advantages of MALDI in the analysis of glycans is its high tolerance to contamination and the ability to analyze samples with a relatively low degree of purification. This technique is also characterized by rapid analysis and high reproducibility, making it suitable for screening studies involving numerous clinical samples. In the context of breast cancer biomarkers, MALDI enables the identification of characteristic glycosylation profiles that can differentiate between cancer and healthy samples [25].

However, the method is not without friction points. One of the main challenges is the difficulty in distinguishing glycan isomers, which may have identical masses but differ in bond configurations. Furthermore, MALDI ionization is subject to “competitive ionization,” where neutral glycans ionize more readily than acidic ones. In breast cancer studies, this is critical because sialylated structures (acidic) are often the most biologically relevant, yet they can be suppressed in the spectrum. Consequently, MALDI is largely a semi-quantitative technique, and signal intensity depends on the ionization properties of individual structures. Therefore, MALDI is often used as a discovery tool, and reliable profiling frequently requires chemical stabilization (e.g., permethylation) and validation using complementary methods [31].

In summary, despite these technical challenges, MALDI-TOF MS remains a key approach in the discovery of new glycoprotein biomarkers. It combines high throughput with the ability to analyze complex glycosylation structures, providing a solid foundation for further translational analyses [32].

### 2.2. Sample Preparation for MALDI Analysis

#### 2.2.1. Sources of Biological Material

In glycoproteomic studies of breast cancer using MALDI, a variety of biological materials are analyzed, depending on the study objective. Serum and plasma are particularly attractive due to the possibility of minimally invasive sampling and the presence of numerous secretory and plasma glycoproteins, whose glycosylation patterns may reflect the presence of cancer. Formalin-fixed, paraffin-embedded (FFPE) tissues represent a valuable source of archival material, enabling the analysis of glycan changes directly in a histopathological context. Breast cancer cell lines, on the other hand, are used in mechanistic studies, allowing for controlled analyses of the effects of specific factors on protein glycosylation [22].

#### 2.2.2. Enrichment of Glycoproteins and/or Glycans

Due to the complexity of biological samples and the wide range of protein concentrations, a key step in sample preparation for MALDI analysis is the enrichment of glycoproteins or glycans. Strategies used include lectin-based affinity chromatography, selective binding of glycoproteins to solid supports, and filtration and precipitation methods. Enrichment reduces interference from highly abundant proteins unrelated to glycosylation and increases the sensitivity of detection of less abundant but biologically relevant glycan structures [13].

#### 2.2.3. Enzymatic Release of N-Glycans

The standard approach to N-glycan analysis is enzymatic release from glycoproteins using the enzyme PNGase F, which hydrolyzes the bond between the asparagine residue and the first monosaccharide of the N-glycan chain. This method allows for the recovery of free N-glycans while maintaining their structural integrity, which is crucial for subsequent mass spectrometric analysis. Enzymatic detachment of N-glycans is widely used in the analysis of both serum and FFPE tissue samples and is one of the most standardized sample preparation steps in glycomics [29,33].

#### 2.2.4. Chemical Modifications

After glycans are released, chemical modifications are often used to improve their analytical properties in MALDI-MS. One of the most commonly used methods is permethylation, which involves methylating the free hydroxyl groups of glycans. This treatment increases the hydrophobicity of the molecules, improves their ionization, and stabilizes sialic acid residues, which are particularly susceptible to fragmentation. Permethylation also facilitates the interpretation of mass spectra, making it an essential step in comparative analyses of glycan profiles in breast cancer biomarker studies [31,34].

#### 2.2.5. The Importance of Sample Preparation Standardization

In summary, proper preparation of biological samples is crucial for the reliability and reproducibility of MALDI analyses in glycoproteomics. The choice of biological material, the enrichment method, the method of glycan release, and the chemical modifications used directly influence the quality of the obtained mass spectra. Therefore, standardization of these steps is essential in research on the identification of glycoprotein biomarkers of breast cancer and their potential clinical application [32].

### 2.3. MALDI Imaging Mass Spectrometry (MALDI-IMS)

Building on the fundamental ionization principles described in Section 2.1, MALDI Imaging Mass Spectrometry (MALDI-IMS) introduces a spatial dimension to the analysis. Instead of generating a single bulk spectrum, the laser raster-scans the tissue section point-by-point in a defined grid. A mass spectrum is recorded for each pixel, allowing for the reconstruction of ‘heat maps’ that visualize the distribution of specific ions across the tissue architecture. This unique capability enables the correlation of molecular data directly with histological features [35]. The use of MALDI-IMS in glycomics enables the study of the distribution of N-glycans directly in tumor tissues, including FFPE preparations. Following enzymatic release of glycans in situ, it is possible to detect specific glycan structures in specific tumor regions. Studies have shown that certain types of glycans exhibit selective accumulation in tumor areas compared to healthy tissue, and their distribution may differ between the tumor core, invasive zone, and surrounding stroma. This spatial analysis provides information that cannot be obtained using classical methods based on tissue homogenization. One of the key advantages of MALDI-IMS is the ability to directly correlate mass spectrometry signals with the histopathological appearance of the tissue. After MS analysis, the same sections or adjacent sections can be stained using histological methods such as H&E, allowing for the assignment of specific mass signals to specific tissue structures. In breast cancer, this allows for the association of specific glycan profiles with tumor areas, pre-invasive lesions, or elements of the tumor microenvironment. This approach increases the biological interpretability of MS data and supports the identification of glycans potentially relevant for diagnosis and prognosis. MALDI-IMS is an advanced tool for analyzing the heterogeneity of breast cancer at the glycosylation level. Combining molecular information with spatial localization allows for the identification of glycans specific to specific tumor regions and their potential association with tumor aggressiveness. In the context of glycoprotein biomarker discovery, this technique provides unique data that can complement classical proteomic and serum analyses [32].

## 3. Strategies for Identifying Glycoprotein Biomarkers

The identification of glycoprotein biomarkers in breast cancer is based on the assumption that the neoplastic process leads to systemic and tissue-specific changes in protein glycosylation [36,37]. Analytical strategies focus on detecting qualitative and quantitative differences in glycan structures between healthy and cancer samples [38]. In this context, mass spectrometry-based discovery approaches are particularly important, as they enable the simultaneous analysis of multiple glycan structures and the identification of characteristic disease signatures [39].

### 3.1. Glycan Profiling

Glycan profiling involves analyzing the complete set of glycan structures present in a given biological material, regardless of their protein carrier. This approach enables the detection of global changes in glycosylation accompanying neoplastic transformation, such as increased N-glycan branching, increased sialylation, or fucosylation. In breast cancer, the observed changes in glycan profiles reflect deregulation of glycan biosynthetic pathways and may constitute a molecular record of processes associated with cancer progression. MALDI-TOF MS is frequently used for this type of analysis due to the ability to rapidly obtain reproducible mass spectra of glycans [29]. A key element of glycan profiling strategies is the comparative analysis of samples from breast cancer patients and healthy individuals. MALDI-MS-based studies have shown that specific N-glycan structures occur with different frequencies or signal intensities in serum and cancer tissues compared to control samples. These differences may relate to both the presence of new glycan structures and changes in the ratios between existing forms. This approach enables the identification of glycan signatures with diagnostic and prognostic potential, particularly in the context of screening and disease monitoring. Glycan profiling is an effective discovery strategy, allowing the identification of changes associated with breast cancer without the need for prior knowledge of the protein carriers of glycans. The resulting patterns can then serve as a starting point for more detailed glycoproteomic analyses aimed at identifying the specific glycoproteins responsible for the observed changes. Combined with MALDI, this approach offers high throughput and translational potential for breast cancer biomarker research [32].

### 3.2. Glycoprotein Analysis (Glycoproteomics)

#### 3.2.1. Identification of Glycan Carrier Proteins

Glycoprotein analysis focuses on identifying specific proteins that undergo aberrant glycosylation in breast cancer. Unlike global glycan profiling, the glycoproteomics approach allows for the association of observed structural changes in glycans with specific protein carriers, increasing their biological and clinical value. Mass spectrometry-based studies, including MALDI, often employ enrichment of glycoproteins or glycopeptides prior to analysis, enabling the detection of less abundant but functionally important proteins associated with cancer processes. This approach has allowed the identification of numerous plasma and tissue glycoproteins whose modified forms are associated with the presence and progression of breast cancer [40].

#### 3.2.2. Glycosylation Site Analysis

An essential element of glycoproteomics is the analysis of glycosylation sites, i.e., the determination of specific amino acid residues to which glycans are attached. Identification of N-glycosylation sites, most often located in the Asn-X-Ser/Thr sequence motif, allows for the assessment of whether neoplastic changes affect not only the glycan structure itself but also the degree of occupancy of individual glycosylation sites [41,42]. In breast cancer, it has been demonstrated that some proteins exhibit altered glycosylation site occupancy or the presence of unusual glycoforms, which may affect their stability, localization, and biological function. Analysis of such changes provides precise molecular information crucial for biomarker identification [43].

#### 3.2.3. The Importance of Glycoproteomic Analysis in the Context of Biomarkers

Combining the identification of carrier proteins with the analysis of their glycosylation sites significantly increases the specificity of potential biomarkers for breast cancer. Instead of assessing the mere presence of a given protein, it is possible to detect specific glycoforms characteristic of cancer cells. This approach is particularly important in translational research, as it allows for a better understanding of the biological mechanisms underlying glycosylation changes and supports the development of more precise diagnostic tools based on MALDI techniques [32,44].

### 3.3. Quantitative and Statistical Approaches

#### 3.3.1. Differential Analysis

Once spectral data are pre-processed (baseline subtraction and peak alignment), differential analysis is performed to identify statistically significant changes. This step moves beyond simple visual inspection to rigorously compare signal intensities between cohorts (e.g., cancer vs. control). Statistical tests are employed to isolate glycan structures whose relative abundance differs significantly, defining a candidate signature [29,45].

#### 3.3.2. Biomarker Classification and Validation Methods

After initial selection of discriminatory features, MALDI data are often analyzed using classification methods such as cluster analysis, principal component analysis, or machine learning algorithms. The goal of these methods is to assess the ability of selected glycans or glycoproteins to discriminate between cancer and control samples. A key element of this stage is biomarker validation, which involves testing classification models on independent data sets and assessing parameters such as sensitivity, specificity, and predictive value. Studies indicate that only a combination of statistical analysis and rigorous validation allows for a reliable assessment of the clinical potential of glycoprotein biomarkers [46,47].

#### 3.3.3. Integration of MS Data with Bioinformatics Analysis

Due to the high complexity of data generated in glycoproteomic studies, integrating mass spectrometry results with bioinformatics tools is becoming increasingly important. This includes assigning glycan structures to specific biosynthetic pathways, analyzing associations with glycosylation enzyme expression, and correlating MS data with clinical and molecular information. This approach enables not only the identification of biomarkers but also a better understanding of the biological mechanisms underlying observed glycosylation changes in breast cancer. Data integration is particularly important in the context of translational research and the development of personalized diagnostic strategies.

#### 3.3.4. The Importance of Quantitative and Statistical Approaches in Biomarker Research

The use of advanced quantitative, statistical, and bioinformatic methods is an essential element of glycoprotein biomarker research. Combined with MALDI-MS techniques, these approaches enable the selection of the most promising biomarker candidates and the assessment of their potential clinical utility in the diagnosis and monitoring of breast cancer [32].

## 4. Glycosylation Changes in Breast Cancer Detected by MALDI

Studies using MALDI-MS techniques have shown that breast cancer is characterized by specific and reproducible changes in protein glycosylation, which can be detected in both cancer tissues and blood samples. Analysis of glycan profiles reveals that neoplastic transformation leads to remodeling of glycan biosynthetic pathways, resulting in the formation of structures distinct from those observed in healthy tissues. These changes are so characteristic that they may constitute molecular signatures of breast cancer identifiable by MALDI methods.

### 4.1. Changes in N-Glycans

#### 4.1.1. Increased Sialylation and Fucosylation

One of the most frequently observed phenomena in N-glycans associated with breast cancer is increased sialylation and fucosylation of glycan structures. MALDI-TOF MS analyses of serum and tumor tissues have revealed an increased proportion of N-glycans containing sialic acid and fucose residues compared to samples from healthy individuals. These changes are interpreted as a result of deregulated glycosylation enzyme activity in tumor cells and are associated with processes such as immune escape, increased cell migration, and disease progression [29]. Increased sialylation of N-glycans detected by MALDI is often associated with a more aggressive tumor phenotype and poorer clinical prognosis. Comparative studies have shown that the degree of sialylation and fucosylation of N-glycans can differentiate not only tumor and control samples but also breast cancer subtypes and disease stages. Such observations highlight the potential of these modifications as components of glycoprotein biomarker panels. However, it is important to note that discrepancies exist in the literature regarding the specific extent of these changes. Variations in reported sialylation levels may stem from differences in sample preparation—specifically, the efficiency of chemical derivatization (e.g., permethylation vs. esterification) used to stabilize sialic acids in MALDI-MS. Therefore, comparing results across studies requires careful consideration of the specific methodological protocols employed [34].

#### 4.1.2. Presence of Multi-Branched Structures

Another characteristic feature of N-glycans in breast cancer is the increased frequency of multi-branched structures, particularly tri- and tetraantennary glycans. MALDI-MS techniques enable the detection of these structures due to their distinctly different molecular weights. The presence of highly branched N-glycans is associated with the overexpression of enzymes responsible for the elongation and branching of glycan chains, which leads to the formation of more complex glycan species in cancer cells. Studies indicate that multi-branched N-glycan structures promote interactions between cancer cells and the tumor microenvironment and may enhance receptor signaling responsible for proliferation and invasion. Their presence in breast cancer samples, detected by MALDI methods, is considered one of the molecular indicators of advanced neoplastic transformation. For this reason, multi-branched N-glycans are being intensively studied as potential prognostic and diagnostic biomarkers [48].

### 4.2. Alterations in O-Glycosylation

#### 4.2.1. Truncated O-Glycan Structures

Characteristic abnormalities in O-glycosylation are observed in breast cancer, including the appearance of truncated O-glycan structures on membrane and secretory glycoproteins. In healthy breast epithelial cells, O-linked glycans are typically formed as longer chains containing galactose and N-acetylglucosamine, as well as their elongated, branched forms. However, in cancer cells, glycan synthesis is usually halted at early stages, resulting in the accumulation of truncated antigens such as Tn (GalNAc-α-Ser/Thr), sialyl-Tn (STn), and other incomplete glycoforms. This tendency to produce short O-glycans arises primarily from the deregulation of glycosyltransferase enzymes responsible for chain elongation and changes in the localization of glycosylation-initiating enzymes. Together, these factors lead to the common ‘truncated O-glycan’ phenotype observed in breast cancer cells [49,50]. Shortened O-glycans are often sialylated in a very simplified form, which further modifies their biological functions and may influence the interactions of cancer cells with the microenvironment. The use of glycomic methods, including mass spectrometry, confirms that these short glycan forms are more frequently present in breast cancer samples than in healthy tissues. It has been described in the literature that, among others, the MUC1 glycoprotein in breast cancers exhibits significantly shortened and highly sialylated O-glycans, which is one of the most frequently reported examples of such an aberration [51,52].

#### 4.2.2. Association with Tumor Aggressiveness

**Figure 2:**
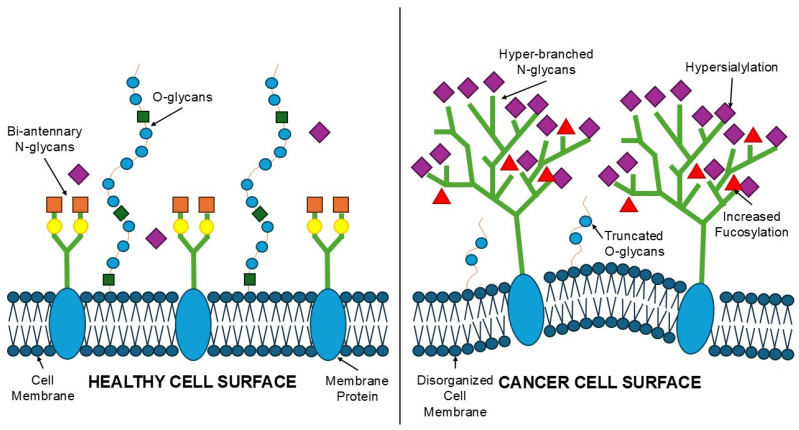
Aberrant Glycosylation Patterns in Cancer Cells Compared to Healthy Tissue. The left panel (Healthy Cell) illustrates a normal lipid bilayer with standard surface proteins displaying simple, bi-antennary N-glycans and extended, stable O-glycan chains. The right panel (cancer cell) depicts a disorganized membrane with characteristic malignant alterations: hyper-branched (multi-antennary) N-glycans, increased density of terminal sialic acid (purple diamonds) and fucose (red triangles), and the presence of truncated O-glycans (Tn/T antigens) (created by the authors).

Alterations in O-glycosylation, particularly the presence of truncated O-linked glycans, are associated with a more aggressive tumor phenotype. Truncated glycans are often found on glycoproteins that play key roles in cell adhesion and signaling, which may promote tumor cell escape from their primary site, facilitate migration, and support invasion. Furthermore, the presence of highly sialylated, short O-linked glycans on the surface of breast cancer cells may modulate immune recognition by immune cells and promote an immunosuppressive environment, further promoting tumor progression and metastasis. Studies also suggest that the expression of specific truncated O-linked antigens may correlate with more advanced clinical features, such as increased proliferation capacity and decreased patient survival. Therefore, truncated O-glycans not only reflect abnormalities in glycan biosynthesis in cancer cells but also have potential as biomarkers related to tumor aggressiveness and clinical outcome (Figure 2) [49,50,51].

### 4.3. Differences Between Molecular Subtypes of Breast Cancer

Breast cancer is a highly biologically heterogeneous disease, reflected in distinctive molecular subtypes with distinct genetic profiles, biological properties, and clinical prognosis. These molecular differences also translate into differential protein glycosylation patterns, which can be detected using mass spectrometry techniques such as MALDI. Analyses of glycan and glycoprotein profiles reveal that individual breast cancer subtypes—Luminal A, Luminal B, HER2-positive, and triple-negative (TNBC)—exhibit distinctive glycosylation features that may have biological and diagnostic significance [53,54].

#### 4.3.1. Luminal A/B

Luminal subtypes, including Luminal A and Luminal B, are characterized by the presence of hormone receptors such as estrogen (ER) and/or progesterone (PR). Many molecular studies have shown that these tumors exhibit a relatively milder clinical phenotype and better prognosis compared to more aggressive subtypes. N-glycan analyses show that luminal subtypes tend to have less pronounced glycosylation changes compared to HER2-positive and TNBC, with lower activity of some branched and fucosylated structures. Furthermore, selected N-glycans with less branching and lower fucosylation may be more typical for these subtypes, reflecting different enzymatic regulations related to glycosylation in ER-positive cells [55].

#### 4.3.2. HER2-Positive

The HER2-positive subtype is characterized by overexpression of the HER2 receptor, which leads to intense proliferative signaling and usually a poorer prognosis than most luminal cases. Glycomic studies have shown that this subtype often exhibits a distinct set of N-glycans, including higher levels of certain complex glycans and alterations in fucosylated and sialylated structures. This distinct glycan profile may reflect specific deregulation of glycosylation enzymes in HER2-positive cells and has the potential to be a diagnostic element differentiating them from other subtypes [55].

#### 4.3.3. Triple-Negative Breast Cancer (TNBC)

TNBC, or triple-negative breast cancer (negative for ER, PR, and HER2), represents a particularly aggressive subtype with high heterogeneity and a less favorable clinical prognosis. Glycosylation studies indicate that TNBC often exhibits unique glycan profiles, including increased levels of structures with a higher proportion of mannose residues and changes in glycosylation, which may be associated with tumor progression and increased invasive capacity. These differences may result from differential regulation of genes involved in glycan biosynthesis in TNBC cells and may be potential markers of subtype [53,55].

#### 4.3.4. Significance of Glycosylation Differences Between Subtypes

Differences in glycosylation between molecular subtypes of breast cancer highlight the fact that glycan changes are not uniform across the breast cancer population. Characteristic N-glycan patterns and their specific modifications may reflect both the biological mechanisms of neoplastic transformation and distinct progression trajectories depending on the subtype. Identification of such differences using MALDI-MS may support not only molecular classification but also the development of more specific diagnostic and prognostic biomarkers tailored to individual breast cancer subtypes [54].

## 5. Clinical Significance of Potential Biomarkers

Identification of glycoprotein biomarkers for breast cancer is of significant clinical importance because changes in glycosylation often appear early in the progression of cancer and may precede visible clinical symptoms. Biomarkers based on specific protein glycoforms can provide more precise information than traditional markers based solely on protein expression levels. MALDI-MS techniques enable the detection of subtle structural differences in glycans, increasing their potential for use in diagnosis, patient stratification, and disease monitoring.

### 5.1. Diagnostics and Early Detection

#### 5.1.1. Serum Biomarkers

Blood serum is an attractive source of glycoprotein biomarkers due to its ease of collection and the possibility of repeated patient monitoring. Studies based on MALDI-TOF MS have shown that the N-glycan profiles present in the serum of breast cancer patients differ significantly from those observed in healthy individuals. Changes identified include, among others, increased sialylation, fucosylation, and the presence of more branched glycan structures, which can act as diagnostic markers. This approach allows for the analysis of entire glycan patterns rather than individual molecules, which increases the sensitivity of disease detection [29].

A critical challenge in clinical diagnostics is differentiating malignant lesions from benign breast conditions such as fibroadenoma or fibrocystic changes. Standard protein markers like CA15-3 often yield false positives due to general inflammation in non-malignant states. However, MALDI-MS profiling has demonstrated the capacity to overcome this limitation. Studies indicate that specific N-glycan alterations—particularly the enrichment of tri- and tetra-antennary structures with specific fucosylation patterns—are significantly more pronounced in invasive breast carcinoma compared to benign tumors. This allows for a more specific ‘molecular fingerprint’ that reduces the risk of misdiagnosis associated with benign breast diseases [29].

Furthermore, it has been shown that specific serum glycoproteins, analyzed at the level of their specific glycoforms, can better reflect the presence of cancer than classical clinical markers. MALDI-MS allows for the detection of such changes in a high-throughput manner, which is important from the perspective of potential screening tests. The use of glycoprotein biomarkers may therefore complement or exceed the effectiveness of established clinical biomarkers in breast cancer.

#### 5.1.2. Noninvasive Diagnostic Strategies

The development of noninvasive diagnostic strategies is one of the key goals of modern oncology, and glycoprotein biomarkers are ideally suited to this trend. Analysis of glycans and glycoproteins in serum, plasma, or other body fluids allows for the detection of neoplastic lesions without the need to collect tissue samples. MALDI-MS techniques, thanks to their high sensitivity and ability to analyze complex biological mixtures, are particularly well-suited for such applications [32]. Studies suggest that the use of glycoprotein biomarker panels can improve the effectiveness of early breast cancer detection, especially when combined with other diagnostic methods. Noninvasive analysis of glycan profiles can also be used to monitor treatment response and detect disease recurrence. Therefore, glycoprotein biomarkers identified by MALDI have the potential to become an important element of future diagnostic and prognostic strategies for breast cancer [22].

### 5.2. Prognostics and Patient Stratification

Protein glycosylation changes observed in breast cancer not only reflect the presence of the disease but also provide prognostic information. A growing number of studies indicate that specific glycan patterns are associated with the clinical course of the disease, making them potential tools for stratifying patients according to the risk of progression and treatment response. The use of MALDI-MS enables the identification of such patterns in a reproducible manner that can be used in clinical trials.

#### 5.2.1. Association of Glycosylation Profiles with Survival and Recurrence

Clinical studies have shown that specific N-glycan profiles present in serum or tumor tissue correlate with overall survival and the risk of breast cancer recurrence. In particular, increased sialylation, fucosylation, and the presence of highly branched N-glycan structures have been associated with poorer prognosis and shorter disease-free survival. MALDI-MS analyses enable the quantitative assessment of these changes, allowing the identification of patients at higher risk of recurrence after primary treatment [29]. Furthermore, differences in glycosylation profiles have been shown to persist or further change during disease progression, making them potential markers for monitoring clinical course. The use of glycan patterns as prognostic biomarkers can support therapeutic decision-making, particularly in cases where classical prognostic factors are unclear. Examples of specific glycoprotein biomarkers identified by MALDI-MS and their diagnostic significance are presented in Table 2.

#### 5.2.2. Predicting Tumor Aggressiveness and Guiding Decisions

Glycosylation aberrations are closely linked to the biological aggressiveness of breast cancer, offering actionable information that traditional markers often miss. While soluble markers like CA15-3 primarily reflect tumor burden, specific glycoforms on membrane proteins—including adhesion molecules and ion channel complexes—reflect the tumor’s functional state: its capacity for invasion and therapy resistance. Studies have shown that tumors with higher metastatic potential are characterized by specific changes, such as an increased presence of truncated O-linked glycans and highly branched N-linked glycans. These changes directly modulate cellular adhesion and interaction with the tumor microenvironment [49].

Consequently, MALDI-MS profiling suggests a potential for patient stratification that could complement standard panels. By identifying these ‘invasive’ glycoforms, future protocols might help distinguish high-risk patients who require aggressive adjuvant therapy from those with indolent disease, even within the same histological subtype. This links molecular membrane biology to potential clinical decision-making, providing a tool for predicting aggressiveness that is mechanistically grounded [51].

### 5.3. Monitoring Treatment and Resistance to Therapy

Protein glycosylation changes observed in breast cancer are not only of significant diagnostic and prognostic importance, but also therapeutic. Accumulating evidence indicates that glycoprotein profiles undergo dynamic changes in response to systemic treatments such as chemotherapy, hormonal therapy, or targeted therapies. Analysis of these changes using MALDI-MS techniques enables tracking the molecular response of the tumor to therapy and identifying mechanisms leading to resistance.

#### 5.3.1. Glycoprotein Changes as Indicators of Treatment Response

Glycoproteomic studies have demonstrated that effective cancer therapy can lead to modifications in glycan profiles in both tumor tissues and circulating serum glycoproteins. Among other things, a reduction in the degree of sialylation and branching of N-glycans was observed in patients responding to treatment, while persistence or exacerbation of these changes was associated with a lack of therapeutic response. The high sensitivity of MALDI-MS allows the detection of such differences at the molecular level, making glycoprotein changes potential indicators of therapeutic efficacy even early in the course of treatment. Furthermore, it has been shown that specific protein glycoforms can appear or disappear during treatment, reflecting adaptive responses of cancer cells to therapeutic pressure. Monitoring these changes can provide information about developing resistance even before clinical signs of disease progression appear. In this context, glycoprotein analysis by MALDI-MS represents a promising tool for supporting the personalization of breast cancer therapy [32].

#### 5.3.2. Potential Therapeutic Targets

Glycosylation aberrations observed in breast cancer not only serve as biomarkers but can also constitute direct therapeutic targets. Changes in the activity of enzymes responsible for glycan synthesis and modification lead to the formation of glycoforms that promote cancer cell survival, migration, and resistance to treatment. The literature has shown that modulation of glycosylation pathways or blocking specific glycan structures can influence the sensitivity of breast cancer cells to therapy [49]. Furthermore, membrane glycoproteins with altered glycosylation may represent attractive targets for targeted therapies or immunotherapy, as their aberrant glycoforms are often specific to cancer cells. Identification of such structures using MALDI-MS allows for the identification of potential therapeutic targets and supports the development of new treatment strategies targeting the unique glycosylation features of breast cancer.

## 6. Critical Limitations and the Validation Gap

While MALDI-MS has democratized access to glycomic profiling, it is crucial to critically assess its limitations to understand why few potential biomarkers have translated into routine clinical practice [58]. A major challenge lies in the distinction between exploratory findings and validated biomarkers. The vast majority of studies reviewed herein represent early-stage discovery phases conducted on relatively small, single-center cohorts. While these studies successfully identify differential glycan patterns, they often lack independent validation on diverse populations, which is essential to rule out bias related to sample collection and processing methods [26].

### 6.1. Technical Reproducibility and Isomeric Resolution

From a technical standpoint, MALDI-TOF MS faces inherent reproducibility challenges. The crystallization process of the matrix and analyte can be non-homogeneous, leading to “sweet spots” and signal intensity variations (shot-to-shot variability). While recent advances in automated spotting and the use of internal standards have improved semi-quantitative capabilities, MALDI remains less robust for absolute quantitation compared to LC-MS/MS or capillary electrophoresis approaches [30]. Furthermore, a significant analytical limitation is the difficulty in distinguishing linkage isomers (e.g., α-2,3 vs. α-2,6 sialylation) based solely on MS1 mass profiles. Since specific linkage types often carry distinct biological meanings in breast cancer progression, the inability of standard MALDI profiling to resolve these isomers without additional derivatization or MS/MS fragmentation represents a limitation in biological specificity [31].

### 6.2. The Validation Gap

Perhaps the most critical bottleneck is the “validation gap.” Many putative biomarkers identified by MALDI are based on global profile changes (e.g., “total fucosylation”). However, clinical utility requires precise, reproducible assays. There is often a discrepancy between the high sensitivity of MALDI in a controlled research setting and its performance in a clinical environment with variable sample quality. Future efforts must focus on bridging this gap by moving from qualitative “fingerprinting” to targeted, quantitative validation of specific glycopeptides using orthogonal methods, ensuring that the identified signatures are biologically robust and not merely artifacts of the analytical workflow.

### 6.3. Standardization and Regulatory Barriers

Even if candidate biomarkers are validated in independent cohorts, a significant hurdle remains in the standardization of workflows across different clinical laboratories [59]. Unlike established automated immunoassays (e.g., Roche or Abbott platforms), MALDI-MS protocols currently lack universal Standard Operating Procedures (SOPs). Variations in matrix preparation, laser energy settings, and incubation times can lead to inter-laboratory discrepancies that are unacceptable in a regulatory context. For MALDI-based glycoprofiling to achieve FDA/EMA approval, the field must move beyond “in-house” protocols toward developing ISO-compliant kits and establishing external quality assessment (EQA) schemes similar to those used in genetic testing. Without this rigorous standardization, even the most promising glycoprotein signatures will remain research tools rather than clinical decision aids [39].

## 7. Future Directions: Integrating Glycoproteomics with Translational Oncology

The future of glycoprotein biomarker research in breast cancer lies at the intersection of technological innovation and clinical application. Although MALDI-based glycoproteomic profiling has already shown significant potential in identifying diagnostic and prognostic biomarkers, its broader impact will depend on integrating glycoproteomic signatures into a comprehensive molecular model that reflects tumor heterogeneity and disease progression. Rather than functioning as an isolated, single analytical tool, in the future, it should be increasingly utilized in multilayer molecular systems.

A particularly promising development is the increasing use of liquid biopsy strategies, which focus on specific circulating components rather than serum [60].

In this context, extracellular vesicles (EVs) have become an attractive source of tumor-derived biomolecules. Unlike plasma proteins, EVs contain membrane proteins, glycoconjugates, nucleic acids, and lipids released directly from tumor cells. This molecular composition provides a more detailed reflection of tumor biology, which can improve the sensitivity of biomarker detection. EVs are not only passive carriers of molecular material but also active factors in tumor progression. In this way, they can contribute to intercellular communication in the tumor microenvironment, the formation of metastatic niches, modulation of the immune system, and, consequently, treatment resistance. Therefore, combining EV isolation strategies with MALDI-based glycoproteomic profiling may be an effective approach to developing minimally invasive diagnostic tools that will allow for capturing dynamic changes in tumor biology [61,62,63].

Another important research direction is understanding the functional consequences of aberrant glycosylation of cancer-associated membrane proteins. Accumulating evidence indicates that glycosylation is not merely a structural modification but a key regulator of protein function and cell signaling. In breast cancer cells, but also elsewhere, altered glycosylation patterns can affect receptor stability, ligand binding, and interactions with the extracellular matrix. This can influence cell adhesion, migration, and invasion. Ion channels provide a particularly interesting example of this phenomenon. Traditionally studied in the context of electrophysiology, voltage-gated sodium channels (VGSCs) are increasingly recognized as regulators of cancer cell motility and metastasis. Their extracellular β-subunits belong to the immunoglobulin family and are highly glycosylated cell adhesion molecules involved in cell–cell and cell–matrix interactions. Experimental studies have shown that these β-subunits can influence tumor cell behavior independently of sodium conductance, highlighting their role as structural and signaling components of the tumor microenvironment. Consequently, aberrant glycosylation of VGSC extracellular domains, including subunits such as SCN3B, can alter adhesion dynamics and promote invasive phenotypes. In this context, MALDI mass spectrometry offers a unique advantage because it enables spatial detection of glycoforms directly in tumor tissues, allowing researchers to map functionally relevant glycosylation changes at the invasive tumor front. To fully realize the translational potential of glycoproteomic data, these findings must also be embedded in a broader multiomic framework [64,65,66,67].

In addition to their diagnostic potential, tumor-associated glycoproteins may also represent promising targets for immunotherapeutic strategies. Many cancer-associated glycoproteins exhibit altered glycosylation patterns, generating tumor-specific antigens capable of eliciting an immune response. These characteristics make extracellular or membrane-bound glycoproteins attractive antigen platforms for the development of therapeutic vaccines. The feasibility of this approach is supported by the rapid development of personalized cancer vaccines, currently undergoing clinical trials. In these strategies, tumor-specific molecular features identified through comprehensive molecular profiling are used to design personalized immunogens capable of stimulating a targeted immune response. Glycoproteomic profiling can complement the discovery of genomic neoantigens by identifying tumor-associated glycoforms. Integrating glycoproteomic data with the development of immunotherapy could therefore open new avenues for personalized cancer treatment. Despite these promising prospects, several challenges must be overcome before glycoproteomic biomarkers can be routinely implemented in clinical practice. Standardization of analytical processes remains crucial, as variability in sample preparation, matrix crystallization, and instrument parameters can affect the reproducibility of results. Future research should therefore focus on developing standardized protocols and quality control strategies suitable for large-scale clinical trials. Equally important will be the validation of candidate biomarkers in independent, multicenter studies to demonstrate both their diagnostic and prognostic value [68,69,70].

To fully realize the translational potential of glycoproteomic data, these findings must also be embedded in a broader multiomic framework. In the complex tumor environment, genomic mutations, metabolic adaptations, and post-translational modifications are primarily responsible for tumor characteristics. Therefore, glycoprotein signatures identified by MALDI-MS should not be interpreted as isolated biomarkers, but rather as functional components of integrated molecular networks. Large-scale multiomic analyses have demonstrated how combining genomic, transcriptomic, proteomic, and epigenomic datasets can reveal oncogenic drivers and pathway-level mechanisms that contribute to tumor progression. Within such an integrated framework, glycoproteomic changes can serve as readout maps of metabolic pathway activity, linking upstream molecular changes with downstream functional consequences. This systems perspective and therapeutic approach can significantly improve clinical interpretation [71].

Beyond its established role as an analytical tool in proteomics and glycomics, MALDI-based mass spectrometry can also be viewed in the broader context of biosensors as a tool for biomarker discovery. Biosensor systems integrate molecular recognition and data interpretation, enabling sensitive and, above all, early detection of disease-related molecules. In this respect, MALDI-MS platforms function as versatile biosensor interfaces, capable of detecting complex biomolecular entities, including glycoproteins and glycans, with high specificity and throughput. Rather than relying on single analytical instruments, mass spectrometry-based technologies are increasingly part of integrated diagnostic ecosystems that combine advanced sample preparation methods, microfluidic technologies, and computational analysis. Recent work on biosensor-based biomarker analysis highlights how such platforms connect molecular discoveries with the clinical application of diagnostic strategies in oncology [72].

## 8. Conclusions

MALDI-MS has proven to be an effective and versatile tool in identifying and characterizing glycoprotein biomarkers associated with breast cancer. By enabling the rapid detection of subtle changes in N- and O-linked glycan structures—including increased sialylation, fucosylation, and the appearance of truncated or branched structures—this technology offers a molecular resolution that transcends standard protein expression analysis. These analyses allow not only for the differentiation of cancer samples from healthy ones but also for the precise refinement of molecular subtypes (Luminal A/B, HER2-positive, TNBC), highlighting the unique value of MALDI in deciphering tumor heterogeneity.

The clinical implications of these findings are profound. Glycosylation profiles detected by MALDI show promising potential in the diagnosis of early breast cancer, prognosis, and therapy monitoring. Importantly, this represents a conceptual shift in biomarker discovery: specific glycan patterns—such as those found on ion channels or adhesion molecules—are directly associated with tumor aggressiveness, risk of recurrence, and treatment response. This link holds promise for more accurate patient stratification and supports the design of personalized therapeutic strategies. Analyses of serum and tissue indicate that these detected glycosylation signatures may evolve into non-invasive markers supporting critical clinical decisions. However, to translate these discoveries into routine clinical practice, the field must address several critical challenges. These steps include: (1) Standardization: Developing rigorous protocols for sample preparation and data acquisition to overcome MALDI’s inherent ionization biases and ensure reproducibility between laboratories. (2) Validation: Bridging the “validation gap” by testing candidate biomarkers in large, independent patient cohorts to confirm their diagnostic and predictive value. (3) Integration: Moving towards a multi-omics approach that combines glycoproteomic data with genomics and transcriptomics for a comprehensive understanding of breast cancer biology. (4) Technological Evolution: Implementing quantitative methods and automating analysis.

Addressing these challenges is crucial for the potential of MALDI-MS to be fully realized, transforming it from a promising discovery tool into a cornerstone of personalized oncology.

## Figures and Tables

**Table 1:** Comparison of analytical techniques used in breast cancer glycoproteomics.

Feature	MALDI-TOF MS	LC-ESI-MS/MS	Lectin Microarrays
Primary Application	Rapid profiling/tissue imaging	Detailed structural sequencing and quantitative proteomics	High-throughput glycan pattern screening
Throughput	High (seconds per sample)	Moderate to low (minutes per sample; chromatography-dependent)	High
Ionization	Predominantly singly charged ions (simplified MS1 spectra)	Multiply charged ions (facilitates MS/MS analysis)	N/A (optical detection)
Fragmentation Strategy	Typically MS1 profiling; optional TOF/TOF fragmentation	Tandem MS with controlled fragmentation (e.g., CID, HCD, ECD, ETD)	Not applicable
Isomer Resolution	Limited at MS1 level; may require derivatization or MS/MS	High (chromatographic separation + MS/MS fragmentation)	Moderate (depends on lectin specificity)
Sample Preparation	Relatively simple; salt-tolerant	More complex; requires chromatographic separation and cleanup	Minimal
Data Complexity	Moderate (pattern-based interpretation)	High (multi-charge states + fragmentation spectra)	Low (intensity heatmaps)
Quantitative Capability	Semi-quantitative (ionization-dependent)	High (label-free or isotope-based quantitation)	Relative intensity-based
Key Advantage	Speed, robustness, and spatial imaging capability	Structural depth and site-specific characterization	Simplicity and rapid comparative screening

**Table 2:** Key glycoprotein biomarkers in breast cancer identified by MALDI-MS and their clinical relevance.

Biomarker Candidate	Biological Source	Glycosylation Alteration	Clinical Relevance & Mechanism	Ref.
MUC1 (CA15-3)	Serum/Tissue	Truncated O-glycans (Tn, Tn antigens)	Indicates neoplastic transformation; promotes immune evasion and metastasis.	[49]
HER2 Receptor	Tissue	Hypersialylation & Fucosylation	Modulates receptor dimerization; associated with resistance to Trastuzumab therapy.	[51]
Total Serum N-glycome	Serum	Increased Sialylation, Fucosylation & Branching	Diagnostic “Fingerprint”: Reflects changes in abundant proteins (e.g., IgG, Haptoglobin). Differentiates cancer patients from healthy controls with higher sensitivity than single markers.	[29]
Haptoglobin glycoforms	Serum	Increased fucosylation and branching of N-glycans	Potential diagnostic biomarker for breast cancer detection (identified by MALDI-FTICR MS glycomic profiling)	[56]
Tissue N-glycan Signatures	FFPE Tissue	High Mannose & Fucosylation (TNBC) vs. Complex/Bisecting (Luminal)	Subtype Differentiation: Distinguishes aggressive Triple-Negative (TNBC) tumors from Luminal A/B subtypes directly from tissue sections.	[55]
EV-derived metabolic signature (lactic acid, serine, glycerophosphocholine, histidine)	Plasma extracellular vesicles	Altered metabolite profile detected by AuM-assisted LDI-TOF MS	Enables differentiation of breast cancer patients from healthy donors and monitoring of treatment response	[57]

## Data Availability

The original contributions presented in this study are included in the article. Further inquiries can be directed to the corresponding authors.

## Abbreviations

CA15-3	Cancer Antigen 15-3
CID	Collision-Induced Dissociation
ECD	Electron Capture Dissociation
ER	Estrogen Receptor
ETD	Electron Transfer Dissociation
EVs	Extracellular Vesicles
EQA	External Quality Assessment
FDA	Food and Drug Administration
FFPE	Formalin-Fixed Paraffin-Embedded
FTICR MS	Fourier Transform Ion Cyclotron Resonance Mass Spectrometry
HCD	Higher-energy Collisional Dissociation
HER2	Human Epidermal Growth Factor Receptor 2
IMS/MALDI-IMS	Matrix-Assisted Laser Desorption/Ionization Imaging Mass Spectrometry
LC-ESI-MS/MS	Liquid Chromatography–Electrospray Ionization Tandem Mass Spectrometry
LC-MS/MS	Liquid Chromatography–Tandem Mass Spectrometry
MALDI	Matrix-Assisted Laser Desorption/Ionization
MALDI-MS	Matrix-Assisted Laser Desorption/Ionization Mass Spectrometry
MALDI-TOF MS	Matrix-Assisted Laser Desorption/Ionization Time-of-Flight Mass Spectrometry
MS	Mass Spectrometry
MUC1	Mucin 1
PR	Progesterone Receptor
SCN3B	Sodium Voltage-Gated Channel Beta Subunit 3
SOP	Standard Operating Procedure
STn	Sialyl-Tn antigen
TNBC	Triple-Negative Breast Cancer
Tn antigen	N-Acetylgalactosamine-α-Ser/Thr antigen
VGSC	Voltage-Gated Sodium Channel
